# Comparison of two minimally invasive restorative techniques in improving the oral health-related quality of life of pregnant women: a six months randomized controlled trial

**DOI:** 10.1186/s12903-021-01581-5

**Published:** 2021-04-30

**Authors:** May M. Adham, Mona K. El Kashlan, Wafaa E. Abdelaziz, Ahmed S. Rashad

**Affiliations:** 1 Department of Pediatric Dentistry and Dental Public Health, Faculty of Dentistry, Alexandria University, Champolion St., Azarita, 21527 Alexandria Egypt; 2 Department of Economics, Faculty of Commerce, Damanhour University, Damanhour, Egypt

**Keywords:** Papacarie-Duo, Atraumatic restorative technique, Minimally invasive caries removal, Pregnant women, Oral health related quality of life, OHIP-14, Egypt

## Abstract

**Background:**

Women tend to delay dental treatment due to misconceptions regarding the safety of dental procedures during pregnancy which may negatively affect their quality of life. Minimally invasive restorative techniques offer alternatives for caries treatment and can improve their oral health-related quality of life (OHRQoL) during this stage.

**Methods:**

A randomized controlled clinical trial was conducted in 2019 and included 162 pregnant women visiting public family health centers in Alexandria, Egypt, with mild to moderate dental pain due to caries. Participants were randomly assigned into Papacarie-Duo group (n = 82) and ART group (n = 80). The outcome variable was percent change in OHRQoL (oral health impact profile, OHIP-14) after 6 months. T test/Mann Whitney U test were used to compare groups and a multivariable linear regression analysis was conducted to evaluate the factors affecting the outcome variable.

**Results:**

A significant reduction (P < 0.002) was noted in OHIP-14 between baseline and 6 months indicating improvement in OHRQoL in the Papacarie-Duo and ART groups (16.26% and 18.91%, P = 0.120 in bivariate analysis). Multiple linear regression revealed significantly greater reduction in OHIP-14 scores in the Papacarie-Duo than the ART group (regression coefficient = 4.03, 95% confidence interval: 0.652, 7.409, P = 0.020).

**Conclusion:**

Minimally invasive restorative techniques, such as ART and chemo-mechanical caries removal using Papacarie- Duo can improve the OHRQoL of pregnant women suffering from mild to moderate pain due to dental caries. Significantly more improvement was noted in the Papacarie-Duo group after adjusting all other variables.

*Trial registration* ID NCT04619264 (https://clinicaltrials.gov/); November 6 2020, retrospective registration. (https://clinicaltrials.gov/ct2/show/NCT04619264?term=NCT04619264&draw=2&rank=1)

## Background

Oral health is important for general health and the quality of life. The global burden of oral diseases is increasing, especially among poor and disadvantaged populations, because access to care is challenged by limited resources allocated to higher priority conditions [1]. In Egypt, most primary health care facilities have insufficient dental materials and equipment due to problems related to infrastructure or availability of spare parts [2].

Atraumatic Restorative Treatment (ART) was developed to treat caries outside dental clinics in remote and under-served areas [3]. Later, ART was included in the Basic Package of Oral Care in public oral health services in Tanzania, South Africa and Latin America because of its low cost and acceptance by patients compared to conventional treatment involving anesthetic injections and drilling [4]. Another non-invasive technique is the chemo-mechanical caries removal using agents to eliminate infected tissue while maintaining healthy tooth structure without pulp irritation or discomfort. Papacarie is one of these chemo-mechanical agents. Developed in 2003, it contains papain, chloramines and toluidine blue salts, which break partially degraded collagen in carious tissue. Its main advantages are the antibacterial properties, biocompatibility and minimally invasive characteristics [5]. Papacarie-Duo, the newest version of Papacarie, was developed in 2011 and has improved properties including extended durability, no need for refrigeration and higher viscosity [6].

Pregnancy may increase the risk of dental caries initiation or progression due to changes in salivary composition, increased acidity of saliva due to episodes of gastric reflux or neglect of oral care [7, 8]. Pregnant women tend to delay dental treatment due to misconceptions about its safety during pregnancy and the adverse effect that may be caused by local anesthesia to the fetus [9]. The resulting dental pain may negatively affect pregnant women' s quality of life and lead to undue stress [10–12]. Moreover, studies evaluating the impact of health-related quality of life on pregnancy outcomes showed that oral diseases were significant factors affecting social and mental well-being [13, 14].

Oral health related quality of life (OHRQoL) is a multidimensional construct that reflects people's comfort when eating, sleeping, and engaging in social interaction, their self-esteem, and their satisfaction with their oral health [15]. The Oral Health Impact Profile (OHIP-14) is one of the most common instruments to assess OHRQoL [16–18].

Provision of dental care to pregnant women from modest socio-economic background in low-resource settings helps ensure their wellbeing and improve their quality of life. This, in turn, reflects on their general health and safety as well as the safety of their newborns. The treatment modalities with the least cost and least anxiety should be implemented to best suit this group. Non-invasive restorative techniques are examples of such modalities for treating dental caries. The aim of the present study was to compare the effect of two minimally invasive caries removal modalities in posterior teeth, Papacarie-Duo and ART followed by glass ionomer restoration on OHRQoL of pregnant women after six months follow-up in Alexandria, Egypt. The null hypothesis was that there would be no difference between using the two modalities on pregnant women’s OHRQoL.

## Methods

### Study design

This study was conducted as part of a randomized, two parallel-arms, controlled clinical trial assessing the impact of the two treatment modalities on dental pain among pregnant women attending family health units/centers in Alexandria, Egypt, from January to October 2019. Ethical approval was obtained from the Research Ethics Committee, Faculty of Dentistry, Alexandria University (IRB 00010556-IORG 0008839) and the approval of the director of each healthcare center was secured to access the obstetrics and gynecology outpatient clinics. Signed written informed consents were obtained after explaining the aim of the study, risks, benefits and confirming confidentiality of responses. The trial was registered at clinicaltrial.gov NCT04619264. Instructions on proper oral hygiene habits were provided to all participants.

### Participants

Pregnant women were eligible to join the study if they were in the first or second trimester of pregnancy, had at least one posterior tooth with occlusal carious dentinal lesion accessible to hand instruments (International Caries Detection and Assessment System score = 5 or 6) [19] and had at least mild dental pain (at least score 5 mm on a 100-mm-long Visual Analogue Scale (VAS) [20]. Pregnant women with acute pulpitis, swelling or fistula, those having severe gingivitis (Gingival Index (GI) score = 3[21]), uncooperative patients and those who refused to participate were excluded from the study.

The study is a secondary analysis of data from a previous research conducted to compare the effectiveness of chemo-mechanical caries removal using Papacarie-Duo and ART in reducing dental pain among pregnant women [22]. The estimated sample size for the primary research was based on assuming 5% alpha error, 20% beta error and comparing the difference between the percentage reporting no pain after chemo-mechanical caries removal (68%) and ART (35%) [23]. The number of participants was calculated to be 160.

### Randomization

Participants were recruited from the prenatal care units in the family health units/centers then randomly allocated to test and control groups using a computer-generated list [24] by the first author in a ratio of 1:1. The allocation sequence was concealed from the primary researcher in sequentially numbered, opaque, sealed envelopes. Examination, intervention and outcome assessment were performed by the same researcher who was trained and calibrated for the assessment of the restoration evaluation criteria (Kappa of intra-examiner agreement = 0.91). All participants received the interventions in the prenatal clinic on the dental chair in the family health units although several clinics were not properly equipped with fully functioning dental chairs. Blinding of participants was not possible due to the difference between the two techniques.

### Interventions

In the Papacarie-Duo group [25], the gel was applied in the cavities after cleaning with wet cotton pellets. When the gel turned cloudy in colour, this indicated the presence of infected tissue and after 40 s the cavity was excavated and the remaining gel was removed. The process was repeated until no change in gel colour was noted. In the ART group [26], excavation was performed using Darby-Perry #220/221, #17 DE (Hu-Friedy, Chicago, USA) after proper cleaning and isolation. Both techniques did not require the use of local anesthesia. All posterior teeth per person that fulfilled the inclusion criteria (International Caries Detection and Assessment System score = 5 or 6) were included and assigned to one of the study groups.

In both groups, cavities were filled with high viscosity glass ionomer cement (GIC) capsules (RIVA Self-cure, SDI Limited, Bayswater, VIC, Australia). A gloved finger was used to apply pressure on the GIC for one minute. Occlusion was checked and excess material was removed [27].

### Outcome assessment

OHRQoL was assessed by the OHIP-14 which includes 14 questions in seven dimensions, two questions for each dimension: functional limitation, physical pain, psychological discomfort, physical disability, psychological disability, social disability and handicap. We used the Arabic version of OHIP-14 which was previously translated and validated [28]. It was assessed at baseline just before caries removal, then after one and six months. Follow-up was set after one month to assess short term outcomes because most participants follow up pregnancy monthly. Six months was the maximum time to assess long term outcomes while ensuring that the participants have not yet given birth so that it would be possible to find them in the same setting. Responses to the OHIP-14 items were on a 5-point Likert scale ranging from never = 0; hardly ever = 1; occasionally = 2; fairly often = 3; to very often = 4. The OHIP-14 score is the sum of the scores of the 14 statements and ranges from 0 to 56 with higher scores indicating higher frequency of negative impact. The outcome variable was the percent change in OHRQoL measured using this formula: [(OHIP-14 after 6 months- OHIP-14 at baseline)/ OHIP-14 at baseline]*100 [15].

The restorations were clinically evaluated based on the retention of the GIC using the ART criteria reported by Loe et al. [29]. Restorations with scores 0, 1 or 7 were considered successful and those receiving scores 2, 3, 4 or 8 were considered failures. Those receiving scores 5, 6 or 9 were excluded [30].

Demographic data (age and education), data about pregnancy-related variables (pregnancy stage and order of pregnancy) and potential confounders (last dental visit, frequency of toothbrushing, perceived state of teeth and gingiva) were collected using the Arabic version of the World Health Organization (WHO) adults questionnaire [31]. Finally, we assessed the number of decayed teeth based on the WHO criteria [32], the dental plaque accumulation and the gingival condition based on Silness and Loe [21, 33] criteria. Individuals who missed follow-up sessions were reached out by text messages and phone calls for rescheduling to avoid attrition. In addition, participants were motivated through oral health education sessions which were delivered during their follow-up visits to ensure continuous engagement with the participants. Participants were asked about dental visits and none of them reported receiving any during the six months follow-up period.

### Statistical analysis

SPSS (Version 24.0, IBM Corp., Armonk, N.Y., USA) was used for data analysis. Quantitative variables were checked for normality using Shapiro Wilks tests, histograms and QQ plots. Significance level was set at p < 0.05. An intention-to-treat analysis was used where patients who were lost to follow up were given the worst OHIP-14 score and restorations were considered as failure. Chi-square and t tests were used to compare baseline characteristics between the two groups. Internal consistency of OHIP-14 items was assessed using Cronbach’a alpha and the overall score was calculated.

T test was used to assess the difference in percent reduction of OHIP-14 scores based on restoration success. Differences in OHIP-14 scores at each time point between the two groups were assessed using independent t test. Mann Whitney U test was used to assess the difference in percent change of each OHIP-14 dimension between groups. Repeated measures ANOVA with Bonferroni correction followed by Post Hoc test was used to assess changes in OHIP-14 scores across time in each group.

**Fig. 1:**
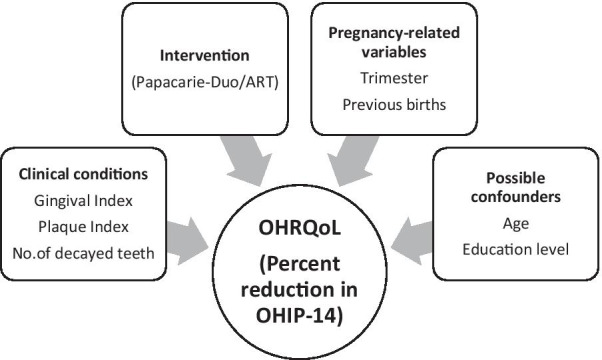
Theoretical framework of the relationship between independent variables and OHRQoL of pregnant women

Multivariable linear regression was conducted to assess the factors affecting percent reduction in OHIP-14 scores. The independent variables in the model were selected based on the framework presented in Fig. 1 [34–37].

## Results

**Fig. 2:**
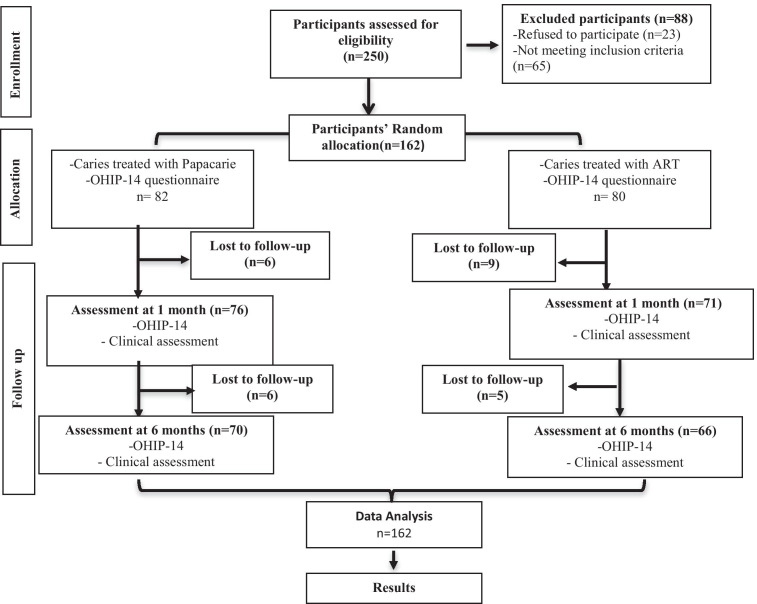
Flow chart

A total of 162 women fulfilled the inclusion criteria and were randomly allocated to the Papacarie-Duo group (n = 82) and the ART group (n = 80). The number of participants lost to follow up at each stage is shown in Fig. 2. The drop-out rate after 6 months was 14.6% and 17.5% respectively.

The mean ± SD age for the Papacarie-Duo and ART group were 27.06 ± 2.82 and 26.3 ± 3.18, P = 0.108. Most participants had secondary education (58.5% and 63.8%, P = 0.744), were in the second trimester (64.6% and 73.8%, P = 0.209) and were pregnant in the second baby (42.7% and 46.3%, P = 0.719, Table 1) with no significance difference between both groups.

**Table 1:** Comparing oral health practices, oral health status, personal and pregnancy profile of the study participants in the two groups (N = 162)

	Papacarie-Duo	ART	P
	Mean (SD)	Mean (SD)	
Age	27.1 (2.82)	26.3 (3.18)	0.108^a^
No. of decayed teeth	2.91 (0.86)	2.71 (0.92)	0.150^a^
Gingival Index	1.54 (0.32)	1.43 (0.29)	0.025^a^*
Plaque Index	1.69 (0.34)	1.65 (0.29)	0.428^a^
Restoration success	68 (82.9%)	63 (78.8%)	0.499^a^
	n (%)	n (%)	
*Education*			0.744^b^
Primary/secondary education	60 (73.2%)	60 (75%)	
University education	22 (26.8%)	20 (25%)	
*Stage of pregnancy*			0.209^b^
First trimester	29 (35.4%)	21 (26.3%)	
Second trimester	53 (64.6%)	59 (73.8%)	
*Order of pregnancy*			0.719^b^
First child	23 (28%)	18 (22.5%)	
Second child	35 (42.7%)	37 (46.3%)	
Third or more	24 (29.3%)	25 (31.3%)	
*Brushing frequency*			0.155^b^
Less than daily	38 (46.3%)	46 (57.5%)	
Daily	44 (53.7%)	34 (42.5%)	
*Last dental visit*			0.308^b^
Within the last year	39 (47.6%)	46 (57.5%)	
More than one year	43 (52.4%)	34 (42.5%)	
*Perceived state of teeth*			0.157^b^
Good/very good	32 (39%)	29 (36.3%)	
Average	34 (41.5%)	29 (36.3%)	
Poor/very poor	16 (19.5%)	22 (27.4%)	

*Statistically significant at p ≤ 0.05

^a^Independent T test

^b^Chi square

There were no statistically significant differences between groups in brushing frequency (P = 0.155), time of last dental visit (P = 0.308) or perceived state of teeth (P = 0.157). Also, there were no significant differences between groups in the number of decayed teeth (P = 0.150), and plaque accumulation (P = 0.428). However, women in the Papacarie-Duo group had significantly greater gingival inflammation than those in the ART group (mean = 1.54 and 1.43, P = 0.025). Restoration success in the Papacarie-Duo and ART groups were 82.9% and 78.8%, respectively (P = 0.499, Table 1).

The Cronbach’s alpha of all OHIP-14 items was 0.89, indicating high internal consistency. Table 2 presents the scores of the OHIP-14 in the two study groups at different time points. There were no differences between groups at baseline (19.51 ± 7.37 and 19.76 ± 7.25, P = 0.828), at one month (16.95 ± 6.41 and 16.65 ± 6.07, P = 0.74) or after six months (16.18 ± 6.25 and 15.73 ± 5.63, P = 0.607). In both groups, the OHIP-14 scores significantly decreased across time (P < 0.001) showing significant difference between successive follow-up intervals, with percent reduction = 16.17% and 18.91% after 6 months and no significant difference between both groups (P = 0.120).

**Table 2:** Comparison of OHIP-14 between groups and across time

	Papacarie-Duo n = 82	ART n = 80	P
Baseline	19.51(7.37)^a^	19.76(7.25)^a^	0.828
1 Month	16.95(6.41)^b^	16.65(6.07)^b^	0.74
6 months	16.18(6.25)^c^	15.73(5.63)^c^	0.607
P value	< 0.001*	< 0.001*	
Percent reduction	16.17(10.16)	18.91(11.4)	0.120

*Statistically significant at p ≤ 0.05

^a,b,c^Different letters denote significant difference between time points within each group

Significantly greater reduction in OHIP-14 score was noted in patients who had successful restorations than patients with failed ones (mean = 19.04 and 11.35, P = 0.004).

Table 3 shows the multivariable linear regression model with the dependent variable being percent reduction in OHIP-14 scores. The model significantly accounted for 11% of the variation in OHIP-14 reduction (adjusted R^2^ = 0.112, F = 3.25, P = 0.001). The type of intervention significantly impacted the reduction in OHIP-14 scores (P = 0.020,). Those in the Papacarie-Duo group had 4.03 greater reduction in OHIP-14 scores than those in ART. None of the other pregnancy factors, clinical factors or confounders were significantly associated with OHIP-14 reduction (P > 0.05). Plaque index was removed from the model due to collinearity with the gingival index.

**Table 3:** Multiple linear regression for factors affecting reduction in OHIP-14 after 6 months

	B(95% CI)	P value
Age	− 0.647 (− 1.361, 0.066)	0.075
*Education level*		
Less than university	2.04 (− 1.701, 5.781)	0.283
University or higher	Reference	
OHIP-14 at baseline	− 0.128 (− 0.488, 0.231)	0.482
Pregnancy related variables		
*Trimester*		
First	1.538 (− 1.972, 5.048)	0.388
Second	Reference	
*Previous births*		
First child	− 1.43 (− 7.266, 4.405)	0.629
Second child	0.384 (− 3.873, 4.641)	0.858
Third or more	Reference	
*Clinical conditions*		
Gingival Index	− 6.829 (− 13.869, 0.211)	0.057
No.of decayed teeth	− 0.9 (− 3.358, 1.557)	0.470
*Intervention*		
Papacarie	4.03 (0.652, 7.409)	0.020*
ART	Reference	

Adjusted R^2^ = 0.112, F = 3.25, p = 0.001. B: regression coefficient, CI: confidence interval, *statistically significant at p ≤ 0.05

## Discussion

Minimally invasive caries removal using Papacarie-Duo and ART significantly improved OHRQoL of pregnant women after one and six months. After adjusting for confounders, Papacarie-Duo group showed greater reduction in OHIP-14 scores. Therefore, the null hypothesis can be rejected.

These findings have implications for the dental care of pregnant women with low or moderate socioeconomic status who suffer from mild to moderate dental pain due to occlusal caries in posterior teeth. The findings can be used to address possible misconceptions about dental treatment and limited access to regular dental care. These factors put them at risk for negative impact on their health and well-being. Keeping good oral health helps ensure safe pregnancy and good quality of life [12]. Such treatment modalities may, thus, reduce inequalities in access to care between pregnant women from different socio-economic levels and ensure the least negative impact on their quality of life.

Our findings agree with previous studies showing how dental care improves the OHRQoL in pregnant women. Musskopf et al. [12] investigated the impact of receiving periodontal treatment on the change of OHRQoL among pregnant women and reported significant reduction in OHIP-14 scores. Retori et al. [38] assessed the association between oral hygiene habits and OHRQoL among 100 pregnant women and showed that tooth brushing > 2 times a day was a protective factor against negative impact on OHRQoL.

This study is probably the first to compare the effect of two minimally invasive caries removal methods on the OHRQoL of pregnant women. There is only one previous study conducted in Brazil [39] that compared the effect of minimally invasive caries removal methods (Carisolv and ART) on the longevity of restorations among pregnant women with no assessment of their OHRQoL. The study reported that both methods were successful which is consistent with the present findings despite differences in the materials used and the length of the follow up.

There is a scarcity of studies assessing the impact of minimally invasive restorative techniques on the OHRQoL in pregnant women and in adults in general. Thus, direct comparison with previous studies is difficult. However, the current results are in agreement with a systematic review reporting significant improvement in the OHRQoL of different population groups after caries treatment [40] and with Paula et al. [41] who reported significant reduction in OHRQoL scores in children after treatment with ART.

In the present study, the OHIP-14 scores at baseline were substantially greater than that of pregnant women in India [34], Brazil [42] and China [43], and relatively higher than those of women in southeastern Brazil [12]. These larger values may possibly suggest the greater impact of dental pain and dental problems on the quality of life of women in the present study. It may also reflect longer duration and higher level of accumulated unmet treatment needs and greater awareness of women regarding the impact of these problems on their quality of life. Further research is needed to assess factors affecting this impact on OHRQoL and why it is more negative than that of women in countries having similar income levels.

The success rate of restorations reported in the present study was similar to that of other studies reporting 100% success rates among pregnant women with chemo-mechanical caries removal agents and 97.6% with ART [39]. The relatively lower success rate in the present study may be attributed to loss to follow up and assigning the worst score to lost cases based on the intention-to-treat analysis. Restoration success had significant impact on OHRQoL improvement in the present study. This indicates that the impact on OHRQoL is mainly related to the effectiveness of the restoration and not just an effect attributed to receiving the treatment regardless of its success.

The present study revealed that besides being effective in caries treatment, Papacarie-Duo had greater impact on OHRQoL of expectant mothers with mild to moderate dental pain. It is important to include patient-reported outcome measures in clinical trials besides clinical and dentist-assessed measures. OHRQoL measures capture the impact of treatment from patients’ perspectives and this is critical for interventions addressing oral health problems that are partly perpetuated by patient behavior or misconceptions such as in the present study.

One of the limitations of this study is related to the short time frame of pregnancy which did not allow a longer follow-up period. There might have been a tendency to report lower impact of oral health because of social desirability bias. In addition, patients with poor oral health and poor expectation may not consider themselves to have poor OHRQoL and consequently report lower scores on OHIP-14. The findings apply to pregnant women and to occlusal caries. In other population groups or types of caries, the findings may not apply and further studies are needed to study these differences. Future studies are needed with larger sample size and longer follow-up periods to assess the long-term effect of minimal invasive caries removal methods.

Papacarie-Duo does not require extensive training for dental health care professionals to be able to use it and does not need sophisticated equipment. It is recommended to integrate this caries removal method as a part of the WHO proposed Basic Package of Oral Care [44] which includes pain relief, preventive, promotional and atraumatic restorative treatments for pregnant women in primary health centers in low- and middle- income countries. Integrating this package with their routine antenatal health care may have great benefits for their oral health and needs to be addressed in future research to help in the control and prevention of dental caries**.**

## Conclusion

Significant improvement in OHRQoL of pregnant women presented with mild and moderate dental pain due to caries was achieved after the use of Papacarie-Duo and ART. Significantly more improvement was noted in the Papacarie-Duo group after adjusting all other variables. Providing dental care to pregnant women from modest socio-economic backgrounds in low-resource settings helps improve their quality of life which reflects on their general health. Including patient-reported outcomes measures like OHRQoL enhances our understanding of the relationship between oral health and patient’s well-being and can be used to inform public policy planning to eradicate oral health disparities.

## Data Availability

The dataset used in this research is available at synapse.org under the title: Impact of minimally invasive restorative techniques on pregnant women oral health related quality of life. Synapse ID: syn23538614. User name: @may.adham.

## Abbreviations

ART: Atraumatic Restorative Treatment
GI: Gingival Index
OHRQoL: Oral Health Related Quality of Life
GIC: Glass Ionomer Cement
SPSS: Statistical package for social sciences
OHIP-14: Oral Health Impact Profile-14
WHO: World Health Organization
